# Validation of a Dutch Risk Score Predicting Poor Outcome in Adults with Bacterial Meningitis in Vietnam and Malawi

**DOI:** 10.1371/journal.pone.0034311

**Published:** 2012-03-28

**Authors:** Ewout S. Schut, Matthijs C. Brouwer, Matthew Scarborough, Nguyen Thi Hoang Mai, Guy E. Thwaites, Jeremy J. Farrar, Johannes B. Reitsma, Diederik van de Beek

**Affiliations:** 1 Department of Neurology, Center for Infection and Immunity Amsterdam (CINIMA), Academic Medical Center, Amsterdam, The Netherlands; 2 Department of Medicine, John Radcliffe Hospital, Oxford, United Kingdom; 3 Oxford University Clinical Research Unit, Hospital for Tropical Diseases, Ho Chi Minh City, Vietnam; 4 Department of Infectious Diseases, Centre for Clinical Infection and Diagnostics Research, Kings College London, London, United Kingdom; 5 Department of Clinical Epidemiology, Biostatistics, and Bioinformatics, Academic Medical Center, Amsterdam, The Netherlands; London School of Hygiene and Tropical Medicine, United Kingdom

## Abstract

We have previously developed and validated a prognostic model to predict the risk for unfavorable outcome in Dutch adults with bacterial meningitis. The aim of the current study was to validate this model in adults with bacterial meningitis from two developing countries, Vietnam and Malawi. Demographic and clinical characteristics of Vietnamese (n = 426), Malawian patients (n = 465) differed substantially from those of Dutch patients (n = 696). The Dutch model underestimated the risk of poor outcome in both Malawi and Vietnam. The discrimination of the original model (*c*-statistic [*c*] 0.84; 95% confidence interval 0.81 to 0.86) fell considerably when re-estimated in the Vietnam cohort (*c* = 0.70) or in the Malawian cohort (*c* = 0.68). Our validation study shows that new prognostic models have to be developed for these countries in a sufficiently large series of unselected patients.

## Introduction

Bacterial meningitis remains an important cause of morbidity and mortality worldwide, even though effective antibiotic therapy is available. [1]–[4] The most common causes are *Streptococcus pneumoniae* and *Neisseria meningitidis*, accounting for 85% of cases in adults. [1]–[4] Fatality rates in patients with meningitis caused by these microorganisms are significant, with rates of 10% and 26% in high-income countries and 15% and 50% in low-income countries. [1], [3], [4] Of surviving patient up to 50% have neurologic deficits, hearing loss, [5] and cognitive deficits. [6], [7]

We previously developed and validated a risk score to predict the risk for unfavorable outcome, using two cohorts of Dutch adults with bacterial meningitis. [3], [8], [9] We first derived a score for the risk for an poor outcome by performing logistic regression analyses of data from a prospective cohort study (n = 696). [3] A key set of independent prognostic variables was selected from 22 potential predictors. A nomogram based on these key variables was constructed to facilitate use in clinical practice. To validate this nomogram, we used data from our randomized controlled trial on adjunctive dexamethasone therapy in adults with bacterial meningitis (n = 301). [9] In the analysis, 6 of 22 variables that are routinely available within 1 hour after admission were robust enough for inclusion in the final risk score: age, heart rate, Glasgow Coma Scale score, cranial nerve palsies, a cerebrospinal fluid leukocyte count less than 1,000 cells/mm^3^, and gram-positive cocci in cerebrospinal fluid Gram's stain. The concordance index for the risk score was 0.84 (95% confidence interval, 0.80 to 0.87) in the original cohort and 0.81 (95% confidence interval, 0.74 to 0.87) in the external validation cohort.

This simple bedside risk score is based on six variables that are routinely collected within an hour of admission and helps physicians to reliably stratify bacterial meningitis patients at initial presentation with respect to the risk for an unfavorable outcome. [8] Risk assessment can be important for physicians because of decisions about the level of care (ward or high-care facility) but may be even more important for informing the patient and his or her relatives. The use of our model may also facilitate the interpretation of future clinical studies in adults with bacterial meningitis because the risk for adverse outcome or the effect of therapeutic interventions is likely to vary in different patient groups.

However, meningitis populations differ substantially depending on the geographic area; the utility of the risk score in other parts of the world is unknown. [1], [2], [4] The aim of the current study was to assess whether this risk score has a similar predictive value in adult meningitis populations from Vietnam and Malawi as previously found in the Netherlands.

## Methods

The Dutch risk model was developed from a series of 696 episodes of adult culture-proven community-acquired bacterial meningitis from a nationwide prospective cohort study. [3], [8], [9] The following six variables were associated with a higher risk for a poor outcome at discharge: higher age, increased heart rate, lower Glasgow Coma Scale score, presence of cranial nerve palsies, a cerebrospinal fluid (CSF) leukocyte count less than 1,000 cells/mm^3^, and Gram-positive cocci in CSF Gram's stain. Poor outcome was defined as a score on the Glasgow Outcome Scale (GOS) of 4 or lower assessed at discharge. The GOS is a well-validated 5-point scale ranging from 1 (dead) to 5 (good recovery). Further details can be found in two previous publications. [3], [8]

The Vietnam cohort was a randomized controlled trial (RCT) of adjunctive dexamethasone therapy for adults in Vietnam. [10] Patients older than 14 years with suspected bacterial meningitis were eligible to enter the study. Inclusion criteria were clinical evidence of meningitis (defined as nuchal rigidity, with elevations in the white-cell count and protein concentration in the CSF) and at least one of the following: bacteria detected in CSF by Gram's or acridine orange stain; a positive CSF latex agglutination test; pathogenic bacteria cultured from either the blood or the CSF; or a clinical history of less than 7 days of illness, with a cloudy CSF, a white-cell count with more than 60% neutrophils, and a ratio of CSF to blood glucose that was less than 50%. The score on the modified Rankin Scale (mRS) was assessed one month after randomization; unfavorable outcome was defined as a score of 1–5, or death. [10] Scores on the GOS were not assessed in this study. Data were complete for all six risk score predictors in 408 of 435 Vietnamese patients (94%). Dexamethasone did not improve the outcome overall in this study, although patients with microbiologically proven disease appeared to benefit. We included patients from both treatment arms.

The Malawi cohort was a RCT of adjunctive dexamethasone therapy for adults in Malawi with bacterial meningitis. [11] Eligible patients were 16 years or older and had suspected meningitis in combination and either cloudy CSF, bacteria in CSF on Gram's staining or a CSF leukocyte count of more than 100 cells/mm^3^ with >50% neutrophils. The score on the GOS was assessed 40 days after randomization; unfavorable outcome was a GOS score of 1–4. Data were complete for all six risk score predictors in 349 of 465 Malawian patients (75%); data concerning cranial nerve palsies were missing in 99 patients (21%). Dexamethasone did not improve the outcome in this study. We included patients from both treatment arms.

Validation was performed by examining calibration, discrimination, and reassessing the logistic regression coefficients of individual predictors of the Dutch model in the Vietnamese and Malawian dataset. The original coefficients of the Dutch logistic regression model were applied to the patients in the validation datasets to calculate the risk score and predicted risk for each patient. The discrimination of the nomogram model in the validation cohorts was quantified by calculating the area under the Receiver operating characteristic (ROC) curve based on a model that only contained the risk score based on the Dutch coefficients. The area under the ROC is also known as the concordance or *c*-statistic. Bootstrap sampling (no. of samples 1999) was used to obtain 95% confidence intervals of the *c*-statistic by taking the 2.5^th^ and 97^th^ percentile value of the bootstrap samples. Calibration or goodness of fit of the original prognostic model was assessed by examining the differences in observed versus predicted probabilities of unfavorable outcome across the range of predicted risk in both the Malawi and Vietnam cohorts. If predictions from the nomogram were consistently too high or too low, we examined whether a change in intercept would improve the calibration. We re-estimated the coefficients of the individual predictors of the nomogram model in the validation cohorts and determined whether these estimates in the validation cohort differed significantly from the original estimates in the Dutch cohort using the independent two-sample Z-test. All analyses were performed using SAS® software version 9.2 (SAS Institute, Cary, North Carolina, USA).

Unfavorable outcome was defined as a score on the GOS of 4 or lower assessed at discharge in The Netherlands and Malawi. In the Vietnam dataset unfavorable outcome was defined as a score on the mRS of 1–5, or death one month after randomization. In the Vietnam dataset additional analyses were performed for mRS scores 0–2 *vs.* 3–5 or death.

The original studies were approved by the research ethics committees of the University of Malawi College of Medicine and the Liverpool School of Tropical Medicine (Malawi study), and the ethics committee of the Hospital for Tropical Diseases (Vietnam study). Informed written consent was given.

## Results

There were many differences in demographic and clinical characteristics between the Dutch cohort (n = 696) and the Vietnamese (n = 426) and Malawian (n = 465) cohorts (Table 1). Patients in Vietnam (median age, 43 years) and Malawi (median age, 33 years) were younger as compared with Dutch patients (median age, 50 years). Antibiotics were administered prior to admission in 55% of Vietnamese patients, and in 25% and 9% of Malawian and Dutch patients respectively (*p*<0.0001). Patients in Vietnam were predominately male (84%) and those in Malawi were almost uniformly HIV-infected (90%). CSF cultures were negative in 49% and 34% patients from Vietnam and Malawi, as compared <1% in the Dutch cohort. The most common causative bacteria were *Streptococcus pneumoniae* in The Netherlands (51%) and Malawi (55%), and *Streptococcus suis* in Vietnam (32%). The percentage of patients with unfavorable outcome varied between cohorts (Netherlands, 34%; Vietnam, 49%; Malawi, 65%).

**Table 1:** Characteristics of adults with bacterial meningitis in the Netherlands, Vietnam, and Malawi.

	Dutch cohort (n = 696)	Vietnamese cohort (n = 435)	P1	Malawian cohort (n = 451)	P2
**Characteristics**					
Age, median years (IQR)	52 (32–67)	41 (17–54)	<0.0001	31 (25–38)	<0.0001
Male sex, n (%)	345 (50)	317 (73)	<0.0001	230 (49)	1.00
Prior antibiotic treatment (n)	64 (9)	238 (55)	<0.0001	123 (26)	<0.0001
HIV infected patients, n (%)	4 (<1)	2 (<1)	1.0	389 (90)	<0.0001
Admission					
Heart rate, median (IQR)	100 (80–114)	92 (80–105)	0.04	100 (90–120)	<0.0002
Diastolic blood pressure, median mm Hg, (IQR)	90 (67–90)	80 (60–80)	<0.0001	70 (60–80)	0.0006
Score on GCS, mean±SD	11±3	12±3	0.01	11±3	0.53
Hemiparesis or monoparesis, n (%)	74 (11)	36 (9)	0.533	21 (6)	0.022
Cranial nerve palsies, n (%)	193 (28)	35 (8)	<0.0001	77 (22)*	0.0002
Cerebrospinal fluid WCC counts					
median (IQR)	3000 (704–8533)	2990 (1000–7450)	0.02	480 (131–1880) (n = 461)	<0.0001
<1000/mm^3^, n (%)	184 (29)	106 (25)	0.161	281 (60)	<0.0001
Results Gram stain, n (%)			<0.0001		<0.0001
Gram positive cocci	320 (49)	181 (42)		237 (54)	
Gram negative cocci	225 (34)	21 (5)		8 (2)	
Other bacteria	22 (3)	19 (4)		15(3)	
Negative	87 (13)	211 (49)		179 (41)	
Causative bacteria, n (%)			<0.0001		<0.0001
*Streptococcus pneumoniae*	352 (51)	50 (12)		254 (55)	
*Streptocooccus suis*	4 (<1)	113 (26)		0	
*Neisseria meningitidis*	257 (37)	11 (3)		14 (3)	
No bacteria cultured	3 (<1)	213 (49)		157 (34)	
Median risk score (IQR)	−0.95 (−2.11 to 0.01)	−1.51 (−2.50 to 0.057)	<0.0001	−0.70 (−1.58 to 0.39)	0.0003
Outcome					
Unfavorable outcome (GOS 1–4 or mRS 1–5), n (%)	237 (34)	210 (49)	<0.0001	296 (66)	<0.0001
Death (GOS = 1), n (%)	143 (21)	28 (11)	<0.0001	249 (55)	<0.0001

Numbers are number/number assessed (percentage) or mean ± standard deviation unless otherwise indicated. Median risk score refers to risk score published in reference [3], P1 denotes P-value for comparison between Dutch and Vietnamese cohorts, P2 denotes P-value for comparison between Dutch and Malawian cohorts, HIV denotes human immunodeficiency virus, BP blood pressure, GCS Glasgow Coma Scale, GOS Glasgow Outcome Scale, mRS modified Ranking Scale, CSF cerebrospinal fluid, WBC white blood cells. * any cranial nerve palsy except VIII.

**Figure 1:**
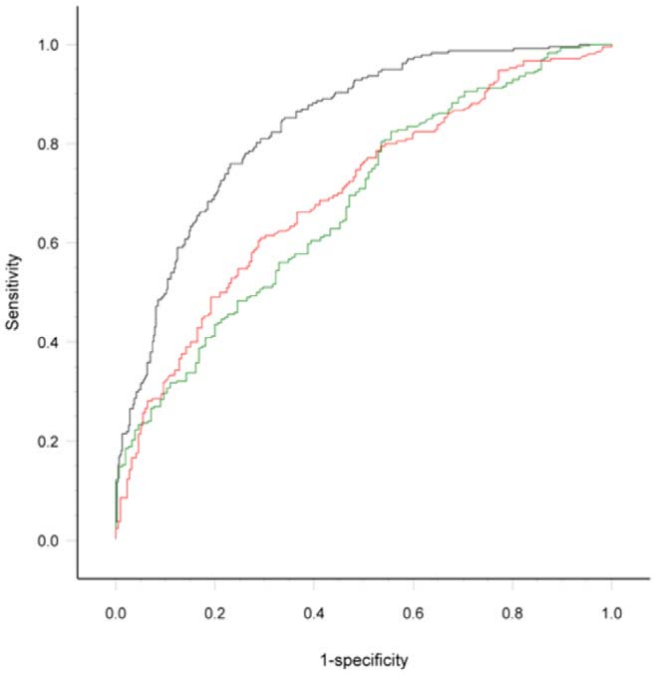
Receiver operating characteristic curves of the final nomogram model in the Netherlands (black), Vietnam (red), and Malawi (green).

The Dutch model (original *c*-statistic = 0.84, 95% CI 0.81–0.86) [1] was less discriminatory in Vietnamese patients (*c* = 0.70, 95% CI 0.65–0.75) and Malawian patients (*c* = 0.68, 95% CI 0.63–0.73). The ROC curves are shown in Figure 1. Calibration showed that the Dutch model underestimated the risk of poor outcome both in Malawi and Vietnam (Table 2). Changing the cut-of for unfavorable outcome in Vietnam (score on mRS 0–2 *vs.* 3–5 or death) did not improve performance of the risk score. The most striking differences between predicted and observed outcome were found in the lower risk groups. Formal goodness of fit tests clearly confirmed the poor calibration: Vietnam, X^2^ = 15.04, degrees of freedom = 4, *p* = 0.005; Malawi, X^2^ = 29.75, degrees of freedom = 4, *p*<0.0001). When analyses were restricted to patients with CSF-culture positive bacterial meningitis, the discriminative power of the models in Vietnam (n = 219; *c* = 0.68, 95% CI 0.60–0.74) and Malawian patients (n = 300; *c* = 0.73, 95% CI 0.67–0.79) remained low. Analyses for patients treated with dexamethasone and placebo showed similar results, with wider confidence intervals.

**Table 2:** Predicted and observed percentage of patients with an unfavorable outcome across the range of predicted risk scores in the validation cohorts for Vietnam and Malawi.

	Predicted and observed percentage of patients with unfavorable outcome					
	Netherlands		Vietnam		Malawi	
Quintiles risk score	Predicted risk %	Observed risk %	Predicted risk %	Observed risk %	Predicted risk %	Observed risk %
First	5	2	6	31	9	43
Second	13	14	13	40	21	64
Third	27	28	27	55	34	63
Fourth	47	52	48	70	54	70
Fifth	75	74	73	82	78	88
All patients	34	34	24	49	39	66

Several coefficients from the original six predictors re-estimated in the Malawian and Vietnamese cohorts differed markedly and statistically significantly from the original value in the Dutch cohort (Table 3). In Vietnam, low CSF WBC count was associated with much lower risk of unfavorable outcome as compared with that in the Netherlands (odds ratio [OR] 1.39, 95%CI 0.85–2.28 *vs.* 3.92, 95%CI 2.51–6.13; *p* = 0.002). Also the impact of type of bacteria seen in the Gram's stain was significantly different (p<0.001) in Vietnam, in particular the presence of other bacteria was associated with a much higher risk of poor outcome than in the Dutch population (Table 3).

**Table 3:** Strength of association between predictors and poor outcome in the Dutch (original nomogram), Malawi, and Vietnamese population.

Prognostic factors	Netherlands	Vietnam	P-value for difference	Malawi	P-value for difference
	OR (95% CI)	OR (95% CI)	P1	OR (95% CI)	P2
Age, yr^a^	1.24 (1.11–1.38)	1.38 (1.21–1.57)	0.23	1.16 (0.93–1.45)	0.60
Heart rate >120 beats/min	2.98 (1.68–5.29)	2.43 (0.79–7.45	0.75	0.99 (0.53–1.87)	0.01
Score on Glasgow coma scale	0.87 (0.82–0.93)	0.86 (0.80–0.93)	0.75	0.79 (0.73–0.85)	0.05
Cranial nerve palsies	2.48 (1.45–4.24)	1.23 (0.46–3.25)	0.22	1.67 (0.91–3.07)	0.34
White-cell count <1000 per mm^3^	3.92 (2.51–6.13)	1.39 (0.85–2.28)	0.002	2.65 (1.70–4.11)	0.22
Gram stain			<0.001		<0.001
Gram positive cocci	1.00 (reference)	1.00 (reference)		1.00 (reference)	
Gram negative cocci	0.27 (0.16–0.45)	0.08 (0.01–0.63)		0.17 (0.02–1.60)	
Other bacteria	0.35 (0.12–1.08)	1.69 (0.56–5.10)		5.17 (1.04–25.66)	
Negative	0.34 (0.18–0.64)	0.73 (0.47–1.14)		1.52 (0.93–2.49)	

a Odds ratios (OR) are calculated in 10-year increments for age; P1 denotes P-value for comparison between Dutch and Vietnamese cohorts; P2 denotes P-value for comparison between Dutch and Malawian cohorts.

Original beta's in the Dutch cohort were: intercept = −0.54; age per 1 year = 0.022; increased heart rate = 1.09; Glasgow Coma Scale score = −0.13; cranial nerve palsy = 0.91; CSF low white cell count = 1.37; gram+ = reference; gram- = −1.29; gram other = −1.04; gram negative = −1.09 The reference category for Gram's stain has been changed in comparison with the original publication.

In Malawi, coefficients of tachycardia (OR 0.99, 95%CI 0.53–1.87 *vs.* 2.97 95%CI 1.67–5.28; *p* = 0.01), score on the Glasgow Coma Scale (OR 0.79, 95%CI 0.73–0.85 *vs.* 0.87 95%CI 0.82–0.93; *p* = 0.05), and bacteria seen in Gram's stain results (p<0.001) differed significantly with those in the Dutch population. In Malawi, no bacteria seen with Gram's stain was associated with the largest increase in risk for a poor outcome, followed by having a negative Gram's stain. In the Dutch population, having Gram positive cocci was associated with the largest increase in risk (see table 3).

We also examined whether inclusion of other characteristics would enhance discriminatory power of the nomogram. In Vietnam, inclusion of prior antibiotic treatment would enhance discriminatory power (OR 1.90, 95% CI 1.18–3.05, *p* = 0.008). In Malawi, inclusion of blood hemoglobulin concentration (OR 0.84, 95% CI 0.78–0.92, *p* = 0.0001) and prior antibiotic treatment (OR 0.37, 95% CI 0.22–0.61, *p* = 0.0001) would enhance discriminatory power.

## Discussion

Our study shows that the Dutch meningitis risk score cannot be used to predict unfavorable outcome in middle- and low-income countries such as Vietnam and Malawi. The discriminatory ability of the Dutch model was substantial reduced in Vietnam and Malawi, indicating that there is more overlap in risk scores between patients with and without an unfavorable outcome. Furthermore, the predicted probabilities of a poor outcome were too low.

Several factors could have contributed to the poorer performance of the Dutch model in these countries. Patients from Malawi were almost uniformly HIV-infected. The high rate of HIV infection has been associated with worse prognosis and is not included in the risk score. [11] Second, the absence of bacteria in a CSF Gram's stain, a factor included in the prognostic score associated with improved prognosis in the Netherlands, might have the opposite effect in Malawi, as it may indicate partially treated pneumococcal meningitis or tuberculous meningitis, which are associated with a much poorer prognosis. [12], [13] Other factors such as suboptimal supportive health care, limited access to health care, and differences in standards of care, *i.e.*, nursing care, fluid management, are also expected to limit the discriminatory power of our model in the Malawian population. Inclusion of blood hemoglobulin concentration, a surrogate marker of malnutrition, and prior antibiotic treatment which was associated with improved prognosis, [4] could enhance discriminatory power of a multivariate prognostic model in Malawi.

In Vietnam, the majority of patients with Gram positive cocci seen on the CSF Gram stain had meningitis due to *S. suis*, the commonest cause of acute bacterial meningitis in southeast Asia. [14] Reported mortality rates are lower (range 3–18%) than in patients with other types of bacterial meningitis. [15]–[17] In contrast with Malawi, prior antibiotic use was associated with unfavorable outcome in Vietnam.

A low CSF WBC count was associated with poor outcome. Clinical studies have shown that lower CSF WBC counts on admission in patients with bacterial meningitis are associated with sepsis and systemic compromise and adverse outcomes later in disease course. [3], [5], [17], [18] Animal studies in a pneumococcal meningitis model showed that lower CSF WBC counts early in disease course were associated with high bacterial load, which correlates with intracranial complications and poor outcome. [19] These experiments also showed that later in disease course, higher CSF WBC counts correlated with high bacterial loads and were associated with poor outcome.

Our study has limitations. Our validation study was based on patients participating in a RCT where they had to meet specific inclusion criteria suggesting that more typical meningitis patients may have been included, and that patients at each end of the disease spectrum (early and very advanced disease) were less represented. This implies that the model is less suited to predict outcome in these patients.

Our results show that the Dutch prognostic model cannot be used accurately to predict outcome in Malawi and Vietnam. In addition, the efficacy of adjunctive dexamethasone varied between populations. The European trial showed a beneficial effect of dexamethasone, [9] while the Malawi trial showed no effect, [11] and the Vietnam trial only showed benefit in proven bacterial meningitis cases. [10] An individual patient data meta-analysis showed no effect when these populations were combined. [20] A follow-up observational study in the Netherlands showed that the introduction of adjunctive dexamethasone improved prognosis from bacterial meningitis, an effect similar to that found in the European RCT. [21] Meningitis populations in high, middle and low income countries appear to be different and prognostic models can not be used interchangeably between these populations. Further studies of prognostic models and underlying pathogenesis must therefore be performed in each population in order to help design rational treatment strategies.
